# Combinational Inhibition of MEK and AKT Synergistically Induces Melanoma Stem Cell Apoptosis and Blocks NRAS Tumor Growth

**DOI:** 10.3390/cells14040248

**Published:** 2025-02-10

**Authors:** Ryyan Alobaidi, Nusrat Islam, Toni Olkey, Yogameenakshi Haribabu, Mathew Shamo, Peter Sykora, Cynthia M. Simbulan-Rosenthal, Dean S. Rosenthal

**Affiliations:** 1 Department of Biochemistry and Molecular & Cellular Biology, Georgetown University School of Medicine, Washington, DC 20057, USA; raa125@georgetown.edu (R.A.); ni98@georgetown.edu (N.I.); tmo49@georgetown.edu (T.O.); yh577@georgetown.edu (Y.H.); mms420@georgetown.edu (M.S.); simbulac@georgetown.edu (C.M.S.-R.); 2 Department of Pathology, King Saud University College of Medicine, Riyadh 11461, Saudi Arabia; 3 Amelia Technologies, LLC, Washington, DC 20001, USA; peters@ameliatechnologies.com

**Keywords:** NRAS, drug resistance, capivasertib, trametinib, xenograft, CRISPR-Cas9

## Abstract

Malignant melanoma is a lethal skin cancer containing melanoma-initiating cells (MICs), implicated in tumorigenesis, invasion, and drug resistance, and characterized by an elevated expression of stem cell markers, including CD133. siRNA knockdown of CD133 has been previously shown to enhance apoptosis induced by the MEK inhibitor trametinib in melanoma cells. This study investigates the underlying mechanisms of CD133’s anti-apoptotic activity in patient-derived BAKP melanoma, harboring the difficult-to-treat NRAS^Q61K^ driver mutation, after CRISPR-Cas9 CD133 knockout or Doxycycline (Dox)-inducible re-expression of CD133. CD133 knockout in BAKP cells increased trametinib-induced apoptosis by reducing anti-apoptotic p-AKT and p-BAD and increasing pro-apoptotic BAX. Conversely, Dox-induced CD133 expression diminished apoptosis in trametinib-treated cells, coincident with elevated p-AKT, p-BAD, and decreased activation of BAX and caspase-3. However, trametinib in combination with pan-AKT inhibitor capivasertib reduced cell survival as measured by XTT viability assays and apoptosis and colony formation assays, independent of CD133 status. CD133 may therefore activate a survival pathway wherein (1) increased AKT phosphorylation and activation induces (2) BAD phosphorylation and inactivation, which (3) decreases BAX activation, and (4) reduces caspases-3 activity and caspase-mediated PARP cleavage, leading to apoptosis suppression and drug resistance in melanoma. In vivo mouse xenograft studies using Dox-inducible melanoma cells revealed increased rates of tumor growth after induction of CD133 expression in trametinib-treated +Dox mice, an effect which was synergistically suppressed by combination treatment. Targeting nodes of the AKT and MAPK survival pathways with trametinib and capivasertib highlights the potential for combination therapies for NRAS-mutant melanoma stem cells for the development of more effective treatments for patients with high-risk melanoma.

## 1. Introduction

Melanoma, the most aggressive form of skin cancer, is ranked as the fourth most common cancer in both men and women, with 100,640 estimated new cases in 2024 and 8290 estimated deaths in the US alone [1]. Based on characteristic mutation signatures (e.g., mutation of two adjacent cytosines (CC) > two adjacent thymines (TT)) and body sites affected, it is estimated that 90% of melanoma cases are linked to UV irradiation, making it a major risk factor for most individuals [2]. Other risk factors include previous genetic predisposition/family history and the presence of atypical nevi [3]. More than 50% of melanoma cases harbor a mutation in the viral *v-raf* murine sarcoma serine/threonine-kinase oncogene homolog B1 (*B-RAF*) proto-oncogene, mimicking an activating phosphorylation by RAS. Roughly 20% have a driver mutation upstream, in the neuroblastoma RAS viral oncogene homolog (*NRAS*) proto-oncogene GTPase-domain [4,5]. NRAS and BRAF mutations both induce constitutive activity, which drives melanomagenesis, through increased proliferation, invasion, metastasis, and drug resistance. Wild-type NRAS, as well as its two homologues, KRAS and HRAS, are normally activated upstream by receptor tyrosine kinases, upon binding to their cognate receptor. Mutations at Q61 cause conformational changes in NRAS, mimicking the active structure. In melanocytes, when epidermal growth factor receptors (EGFRs) on the cell membrane bind their specific ligands (e.g., CKIT or EGF), kinase cascades, including the mitogen-activated protein kinase (MAPK) pathway, are initiated through the binding, phosphorylation, and activation of BRAF by NRAS at two sites flanking V600. V600E mutations mimic the negative charge on phospho-BRAF, causing constitutive activation. Thus, NRAS and BRAF, adjacent nodes in the MAPK pathway, are deregulated by activating mutations, showing the importance of this pathway in melanocyte-derived cells. Downstream of RAF, the MAPK pathway includes the MAP/ERK kinases (MEK1 and MEK2), which are phosphorylated by activated BRAF. MEK1 and MEK2, in turn, phosphorylate and activate extracellular-regulated kinases (ERK1 and ERK2), which then phosphorylate ribosomal S6 kinase (RSK), a 90 kDa family of Ser/Thr kinases which regulate diverse cellular processes. Both RSK and ERKs are released from their anchoring proteins, translocate to the nucleus, and activate transcription factors that promote cell proliferation, metastasis, and survival. Key mutations in either BRAF^V600^ or NRAS^Q61^ continuously activate this pathway, serving as oncogenic drivers in melanoma [6].

When diagnosed at early, fully localized stages (stage 0, I, or II), surgical removal is the most common treatment and has a 99% cure rate. Treatment is dependent on the type of driver mutation for advanced stages (III–IV). Targeted therapies such as BRAF inhibitors (BRAFis), including dabrafenib and vemurafenib, constitute a major advance in the treatment of BRAF-mutant melanoma, although resistance develops quickly. The combination of BRAFi and MEK inhibitors (MEKis) has provided modest improvements in overall survival (OS) [7]. However, due to its high affinity for guanosine triphosphate (GTP) and relatively unstructured character, no drugs binding mutant NRAS have yet proven to be effective. Immunotherapy is the current standard-of-care for NRAS-mutant melanoma, but the efficacy of this type of treatment is inconsistent [8]. While trametinib, an FDA-approved targeted MEKi therapeutic for melanoma has improved the OS of patients with melanoma, most tumors with *NRAS* mutations are, at best, only partially responsive to MEKi-based therapy alone, and the development of resistance remains a challenge. Other options, including combination therapies with MEKi and pan-RAF inhibitors [9], are undergoing clinical trials, although these combinations have not yet shown any significant increase in the OS [10]. With limited treatment options and high relapse rates for melanoma, investigation into the role of alternative pathways in melanoma progression is urgently needed, with the goal of finding novel therapeutic targets. 

Potentially appealing targets include those expressed in human cancer stem cells (CSCs), sub-populations which can self-renew, differentiate, and initiate tumors and are responsible for tumor recurrence and chemoresistance. CSCs in different cancer types and biomarkers for these cells have been identified. CD133, also known as PROM1, is one of the most common markers for the detection and isolation of CSCs [11,12]. It was first identified as a marker for brain CSCs [13,14] and has now been reported in other cancers including those of the prostate [15], lung [16], and stomach [17]. Additionally, CD133+, but not CD133- cells, isolated from patients with colorectal cancer are able to initiate tumors in mice after serial transplantation, with histopathological features similar to those of parent tumors [18,19]. Similarly, CD133 was found to be a CSC marker for hepatocellular carcinoma (HCC) in which cell proliferation was higher in CD133+ cells compared to CD133- cells, and in vivo experiments showed that CD133+ cells initiate significantly larger tumors [20]. Resistance to treatment was also reported in CD133+ HCC [21].

CD133 is a pentaspan transmembrane glycoprotein with an extracellular N-terminal domain and an intracellular C-terminal domain [22]. Localized to chromosome 4, the *CD133* gene has 37 exons and five alternate promoter regions, two of which are regulated by CpG methylation status. Due in part to these promotors, seven spliced mRNA forms with potential structural variants have been observed [23]. Since it was first discovered as a hematopoietic stem and progenitor cell marker [24], the physiological roles of CD133 still remain to be clarified. CD133 localization at the plasma membrane indicates a role in plasma membrane organization, since it binds to plasma membrane cholesterol and enhances membrane stability [25]. CD133-deficient mice revealed photoreceptor cell degeneration and vision impairment but were viable and fertile, with lifespans within normal ranges [26]. Increasing evidence has since shown that CD133 not only functions as a biomarker, but also plays roles in cell self-renewal, metabolism, differentiation, tumorigenesis, metastasis, apoptosis, autophagy, and regeneration. 

We showed that expression of CD133 is associated with drug resistance in melanoma cells. CD133 knockout by CRISPR-Cas9 significantly decreases invasion, metastasis, and chemoresistance in melanoma. We further delineated a survival pathway activated by CD133 where (1) increased AKT phosphorylation and activation induces (2) BAD phosphorylation and inactivation, (3) decreases BAX activation, and (4) reduces caspases-3 and -9 activity and caspase-mediated PARP cleavage, leading to apoptosis suppression and drug resistance in melanoma [5]. The current study therefore aimed to target CD133+ NRAS-mutant melanoma stem cells and the tumors derived from them with capivasertib, a pan-AKT inhibitor which has shown cytotoxicity against other human cancers, in order to increase the efficacy of trametinib by simultaneously inhibiting both AKT and the MAPK pathway in resistant cells. Capivasertib is an attractive therapy, as it is an orally active, potent pan-AKT kinase inhibitor [27]. 

## 2. Materials and Methods

### 2.1. Cells

Human melanoma cell lines BAKP (NRAS^Q61K^) and POT (NRAS^Q61R^) were maintained in Iscove’s Modified Dulbecco’s Medium (IMDM, ThermoFisher Scientific, Waltham, MA, USA) supplemented with 10% FBS and 1% penicillin–streptomycin in a 37 °C 5% CO_2_ incubator. NRAS mutations were verified by Sanger sequencing, and the expression of melanoma markers MART1 and S100 was verified by flow cytometry, as previously described [28]. The patient-derived de-linked human melanoma cell lines BAKP and POT that were used in this study were a kind gift from Dr. John Zapas (Maryland Melanoma Center) and Maryam AbdusSamad. 

### 2.2. CRISPR-Cas9 Deletion of CD133

Three different sgRNA sequences specific to the CD133 exon 1 in lentiviral vector pLenti-U6-sgRNA-SFFV-Cas9-2A-Puro (Addgene.org, Watertown, MA, USA) were packaged and transduced into melanoma cells as previously described [28]. Briefly, for CRISPR-Cas9 knockout (KO), HEK293FT cells were transfected with pLenti-U6-sgRNA-SFFV-Cas9-2A-Puro plasmid containing individual signal–guide sequences (sgRNA) beginning at either 8, 69, or 205 bp downstream of the start of the CD133 coding sequence, within the first exon of CD133. Lentivirus packaging plasmids were transfected into HEK 293FT cells using Lipofectamine LTX (ThermoFisher Scientific, Waltham, MA, USA). Lentivirus released in media after 48 h was then used to transduce melanoma cells, followed by selection for 5 days with puromycin and the isolation of pooled clones, which were subjected to PCR analysis. Alterations of exon 1 were assessed by PCR amplification followed by NGS sequencing to detect allelic or frameshift mutations (%) at CRISPR target sites T1, T2, and T3 [28].

### 2.3. Generation of Doxycycline (Dox)-Inducible Cells 

To generate Dox-inducible lentivirus that can induce CD133 expression, HEK293FT packaging cells were co-transfected with pLenti-CMV-rtTA3 Blast (Addgene, Watertown, MA, USA) and psPAX2 and pMD2.G (Addgene), with or without pLV-EGFP/Neo-TRE3G-CD133 (VectorBuilder Inc., Chicago, IL, USA) using Lipofectamine LTX (ThermoFisher Sci, Waltham, MA, USA). Cells were incubated for 48 h to generate lentivirus. Viral supernatants (MOI = 1) were then used to transduce melanoma cells in 6-well plates. After 24 h, transduced cells were selected with blasticidin (40 µg/mL) and geneticin (1 mg/mL) for 10 days, as previously described [28]. Cells were induced to express CD133 by incubation with 1 μg/mL Dox. 

### 2.4. Quantitative Reverse Transcription PCR (qRT-PCR)

Total RNA was extracted from cell pellets using Trizol Reagent (Gibco BRL, Grand Island, NY, USA) and subjected to qRT–PCR by standard protocols with a two-step reverse transcription PCR (Invitrogen, Waltham, MA, USA), 1 µg RNA, and specific primers listed below. cDNA synthesis was carried out using a Verso cDNA synthesis kit (ThermoFisher Sci, Waltham, MA, USA).

CD133 forward: 5′-CCC GGG GCT GCT GTT TAT A

CD133 reverse: 5′-ATC ACC AAC AGG GAG ATT G

### 2.5. Immunoblot Analysis

Total cell lysates (30 μg per lane) were subjected to SDS-PAGE electrophoresis, and the proteins were transferred to nitrocellulose membranes according to standard procedures. To verify protein transfer and equal loading, the membranes were stained with Ponceau S (0.1%). The membranes were then incubated with antibodies to CD133 (Miltenyi Biotec, Auburn, CA, USA), p-MEK, p-BAD (BioLegend, San Diego, CA, USA), p-ERK, active caspase 3 (Cell Signaling Technology, Danvers, MA, USA), and p-GSK-3β. Anti-β-actin (ProteinTech, Rosemont, IL, USA) was used as the loading control. Immunoblots were stripped of antibodies and sequentially re-probed with up to 3 other antibodies and β-actin. Detection of immune complexes was performed by incubation with horseradish peroxidase-conjugated antibodies to mouse or rabbit IgG (1:3000), followed by enhanced chemiluminescence (ECL; Pierce, Rockford, IL, USA) and imaging in an Amersham Imager 600 (GE Healthcare, Marlborough, MA, USA). 

### 2.6. Drug Treatment and Cell Viability Assays

Cells were seeded in 96-well plates (5 × 10^3^ cells/well), and triplicate wells were exposed to trametinib (100 nM) and capivasertib (1 μM), alone or in combination for 48 h. Cells were treated for 48 h based on initial time course experiments showing maximal effects at that time point [28]. In another time course experiment, cells were treated for 8 h, 24 h, or 48 h with trametinib, alone or in combination with capivasertib, followed by immunoblot analysis with an antibody to the active cleaved form of caspase 3, an apoptosis marker. These results showed no activation, weak activation, and robust proteolytic activation of caspase 3 at 8 h, 24, h, and at 48 h, respectively (Supplementary Figure S2). A dose of 100 nM trametinib was used, since this was the LD40 for trametinib in BAKP melanoma cells as determined in earlier dose–response experiments [29]. Further, a significant dose-dependent decrease in cell viability has previously been shown in this dose range for other tumor cells, such as glioma cells treated with increasing doses from 2 nM to 200 nM trametinib [30]. A dose of 1 μM capivasertib was used based on other studies, such as one showing efficacy of 0.75 to 2 µM capivasertib in combination treatments in prostate cancer cell lines [31]. Drugs were dissolved and diluted in the same final volume of DMSO (vehicle control) in order to achieve a final concentration of 0.2% DMSO in the culture medium. Likewise, control cells were exposed to 0.2% DMSO alone. To determine the effects of drugs on cell viability, a colorimetric XTT assay was used (Biotium, Inc., Fremont, CA, USA) to assess metabolic reduction of XTT by viable cells. Absorbance was measured hourly for 3 h after XTT reagent addition, using an EnSpire multimode plate reader (Perkin Elmer, Waltham, MA, USA) set at 450/650 nm to obtain a slope of A_450_/min. Each plate included wells with the drug-treated or control cells in triplicate, as well as 6 replicates each of an increasing number of cells in medium with 0.2% DMSO to generate a standard curve of A_450_/min vs. cell number. The standard curve was then used to assess % cell viability. 

To validate the results of the XTT cell viability assay, the Sytox-Blue (ThermoFisher Scientific, Waltham, MA, USA) dye exclusion assay was also carried out as an alternative cell viability assay. Control and treated cells were incubated for 15 min with Sytox Blue to detect the loss of intact cell membranes, allowing entry of the dye in nonviable cells, followed by flow cytometric analyses on a FACStar Plus cytometer (BD PharMingen, Franklin Lakes, NJ, USA). The data in each figure represent the mean ± SEM of each set of triplicates from a representative experiment.

### 2.7. Apoptosis Assays: Annexin V-APC/Sytox Blue Staining and Flow Cytometry

After a 48 h treatment of cells with trametinib, capivasertib, or their combination, both floating and attached cells were pooled, trypsinized, and washed with ice-cold phosphate-buffered saline. The cells were then incubated in the dark for 15 min at room temperature with 100 μL of Annexin V-APC (BD Bioscience, Franklin Lakes, NJ, USA) for the detection and quantification of phosphatidylserine on the surface of early-apoptotic cells. Sytox Blue (ThermoFisher Scientific) was also added to the dye mix to detect the loss of intact cell membranes in late-apoptotic cells. The cells were then subjected to flow cytometric analyses on a FACStar Plus cytometer (BD PharMingen, Franklin Lakes, NJ, USA; Georgetown Lombardi Comprehensive Cancer Center Flow Cytometry & Cell Sorting Shared Resource). The flow cytometry analyses allowed the quantification of the % of viable, early-apoptotic, and late-apoptotic cells, as indicated in the lower left, lower right, and upper right quadrants, respectively, of the dot plots, as previously described [32].

### 2.8. Immunofluorescence Staining and Imaging

The cells were fixed with 4% paraformaldehyde, incubated in Superblock (Thermo Fisher Scientific) for 1 h, followed by incubation in a humid chamber at 4 °C overnight with primary antibodies against CD133 (Miltenyi Biotec, Gaithersburg, MD, USA) and the cleaved active form of caspase 3 (Cell Signaling Technology, Danvers, MA, USA). After incubation for 2 h with secondary antibodies Alexa Fluor 488-conjugated goat anti-mouse IgG (Abcam, Cambridge, UK) or Alexa Fluor 594-conjugated goat anti-rabbit IgG (ThermoFisher Scientific), the slides were mounted with ProLong Diamond Antifade (Invitrogen, Waltham, MA, USA), and images were captured with a DP75 digital camera attached to an Olympus immunofluorescence microscope with CellSens Standard version 1.13 imaging software (Evident, Tokyo, Japan). For mitochondrial membrane potential assays, after 48 h of treatment, BAKP cells were subjected to an assay detecting changes in mitochondrial membrane potential via 30 min incubation at 37 °C with tetramethylrhodamine methyl ester (TMRM; Invitrogen), followed by immunofluorescence microscopy. Immunofluorescent images of viable cells showed red fluorescent mitochondrial staining, whereas apoptotic cells showed a decrease in mitochondrial fluorescence.

### 2.9. Clonogenic Assays 

The cells were seeded and exposed to Dox for 24 h and then to capivasertib, trametinib, or the combination of capivasertib + trametinib for 48 h. The cells were collected and counted with an Eve automatic cell counter; then, 1000, 500, and 250 cells were seeded and incubated in 6-well plates for 12 days. The colonies were stained with crystal violet and counted.

### 2.10. Mouse Xenografting 

All animal experiments were performed following approved protocols of the Georgetown University Institutional Animal Use and Care Committee (protocol/project identification number 2016-1218). A week prior to xenografting, athymic *NCr*-*nu/nu* 6-week-old male mice (Harlan Laboratories, Indianapolis, IN, USA) were acclimated to the Georgetown University Division of Comparative Medicine (DCM). BAKP Dox-inducible cells were injected subcutaneously using a 20-gauge syringe, after resuspension in Matrigel matrix (Corning, NY, USA), into the hind flanks of athymic mice, which were fed with either regular or Dox-supplemented feed. Tumor growth in mice was monitored with calipers using the following formula: tumor volume = 1/2 length × width^2^. When tumor volumes reached 100 mm^3^, drug treatment was administered by oral gavage with either vehicle emulsion control, trametinib (3 mg/kg/qd), capivasertib (100 mg/kg/qod), alone or in combination, according to standard DCM procedures. Drug efficacy was monitored daily, and adverse effects were assessed by loss of weight and necropsy (*n* = 5 per treatment group). 

### 2.11. Statistical Analysis

GraphPad Prism9 (GraphPad, San Diego, CA, USA) was used for all statistical analyses. Experiments were performed in biological triplicates; representative data from three independent experiments are presented in this paper. Two group comparisons between the control and test samples were performed by standardized two-tailed Student *t*-tests. The error bars shown on graphs are ± SEM. *p* < 0.05 was considered significant; *, **, ***, and **** represent *p* < 0.05, *p* < 0.01, *p* < 0.001, and *p* < 0.0001, respectively. Bliss independence [33] for survival, apoptosis, and tumor growth was calculated from monotherapies for capivasertib and trametinib and the theoretical fractional response (Y axis) of the combination of two drugs (calculated as the sum of the two fractional responses minus their product), divided by the observed response to a combination of capivasertib and trametinib = (F_C_ + F_T_ − F_C_ × F_T_)/F_CT_, as described in [34]. The combination index (CI) for each dose is shown in Supplementary Tables S1–S4.

## 3. Results

### 3.1. CRISPR-Cas9 KO of CD133 in BAKP Melanoma Cells Increases Trametinib-Induced Apoptosis via Downregulation of Pro-Survival p-BAD and p-AKT

**Figure 1:**
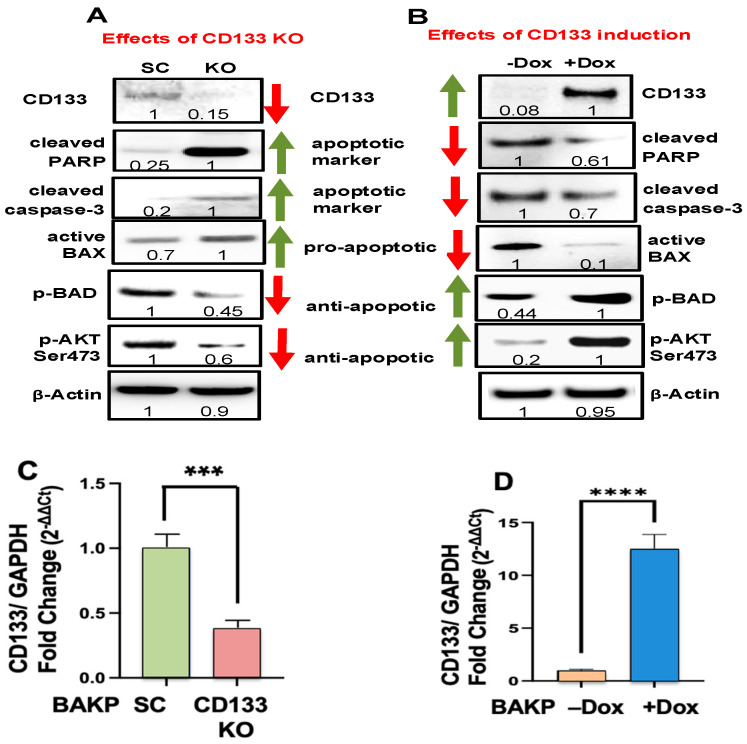
(**A**) CD133 CRISPR-Cas9 KO increases trametinib-induced apoptosis whereas (**B**) Doxycycline (Dox)-induced CD133 expression in BAKP cells decreases apoptosis (BAX activation, PARP cleavage, and caspase-3 activation) following trametinib treatment, stabilized by the upregulation of pro-survival pAKT and pBAD in CD133-expressing cells. The cells were incubated with 100 nM trametinib for 48 h, followed by immunoblot analysis with antibodies to cleaved active caspase 3 and its substrate—cleaved PARP, the pro-apoptotic active form of Bax, the anti-apoptotic phosphorylated form of BAD (p-BAD), and the pro-survival phosphorylated active form of AKT (p-AKT Ser473). After normalizing to β-actin, a densitometric analysis comparing the intensities of protein bands relative to bands with the highest intensities is shown in the immunoblots. Scans of whole-gel immunoblots for all the figures are shown in “Supplementary Materials” (Figure S1). (**C**,**D**) Knockdown of CD133 expression in BAKP CD133-KO cells (**C**) and upregulation of CD133 expression in inducible BAKP cells in the presence of Dox (+Dox; (**D**)), as verified by qRT-PCR analysis. *p* < 0.05 was considered significant; *** and **** represent *p* < 0.001, and *p* < 0.0001, respectively.

We explored potential mechanisms whereby CD133 suppresses trametinib-induced apoptosis in order to promote melanoma cell survival, first by CRISPR-Cas9-mediated knockout of CD133 in BAKP melanoma cells. CD133-KO and control scrambled BAKP-SC cells were incubated with 100 nM trametinib for 48 h, followed by immunoblot analysis with antibodies to CD133 and then pro- and anti-apoptotic markers. In response to trametinib treatment, apoptosis was significantly increased in the BAKP CD133-KO cells compared to the control BAKP-SC cells (Figure 1A), as evidenced by five-fold higher levels of the cleaved active form of caspase 3 and its substrate PARP, as well as the active form of pro-apoptotic BAX, a protein essential for the initial activation of the apoptotic caspase cascade. CD133 knockdown in BAKP CD133-KO cells was verified at the protein and RNA levels by immunoblot analysis (Figure 1A) and qRT-PCR (Figure 1C), respectively. CD133 KO in BAKP melanoma cells can therefore sensitize cells to trametinib-induced apoptosis. This may be partially attributable to the diminished activation of the pro-survival protein kinase B or AKT (p-AKT) and the consequent reduction in the levels of p-BAD, its substrate, in CD133-depleted KO cells.

### 3.2. Dox-Induced CD133 Activates AKT and Suppresses Trametinib-Induced Apoptosis

Dox-inducible BAKP cells, with barely detectable CD133, were treated with Dox to induce CD133 expression and then incubated with 100 nM trametinib for 48 h. Differences in the levels of pro- and anti-apoptotic markers were then examined by immunoblot analysis. Coincident with a robust increase in CD133 protein (Figure 1B) and RNA (Figure 1D), Dox-treated BAKP melanoma cells exhibited a 40% reduction in pro-apoptotic markers, including cleaved active caspases-3, cleaved PARP, and active BAX, indicative of the suppression of caspase 3-mediated apoptosis by CD133 in trametinib-treated cells (Figure 1B). In contrast, anti-apoptotic markers p-BAD and p-AKT were substantially enhanced in Dox-induced CD133+ cells. These results together suggest that, since the MAPK survival pathway was blocked by trametinib, CD133 could suppress apoptosis and promote resistance to trametinib by activating an AKT/phospho-BAD survival pathway in human melanoma cells. Increased cell survival and trametinib resistance in CD133+ melanoma stem cells may be facilitated, at least in part, by the activation of this alternative survival pathway. These results have been validated in another Dox-inducible melanoma cell line, POT [35], indicating that these responses are not cell line-specific.

### 3.3. The Simultaneous Inhibition of MAPK and AKT Pathways by a Combination of Trametinib+ Capivasertib Is Significantly More Cytotoxic in Different NRAS Melanoma Cell Lines

**Figure 2:**
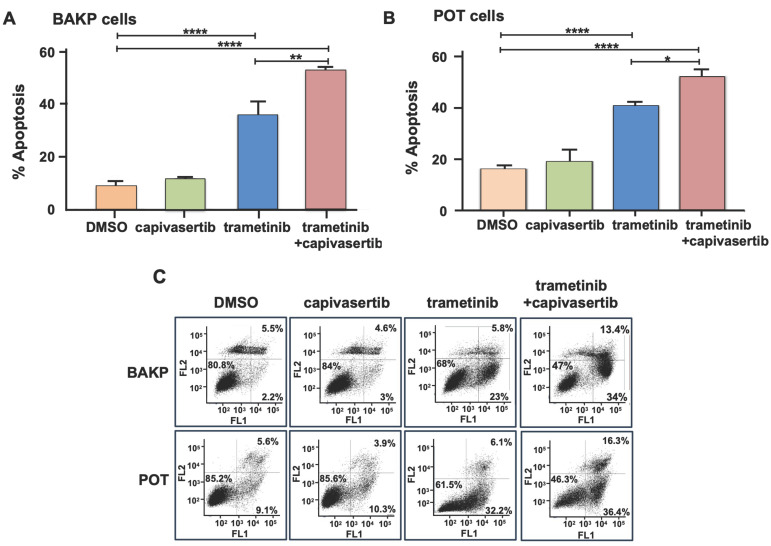

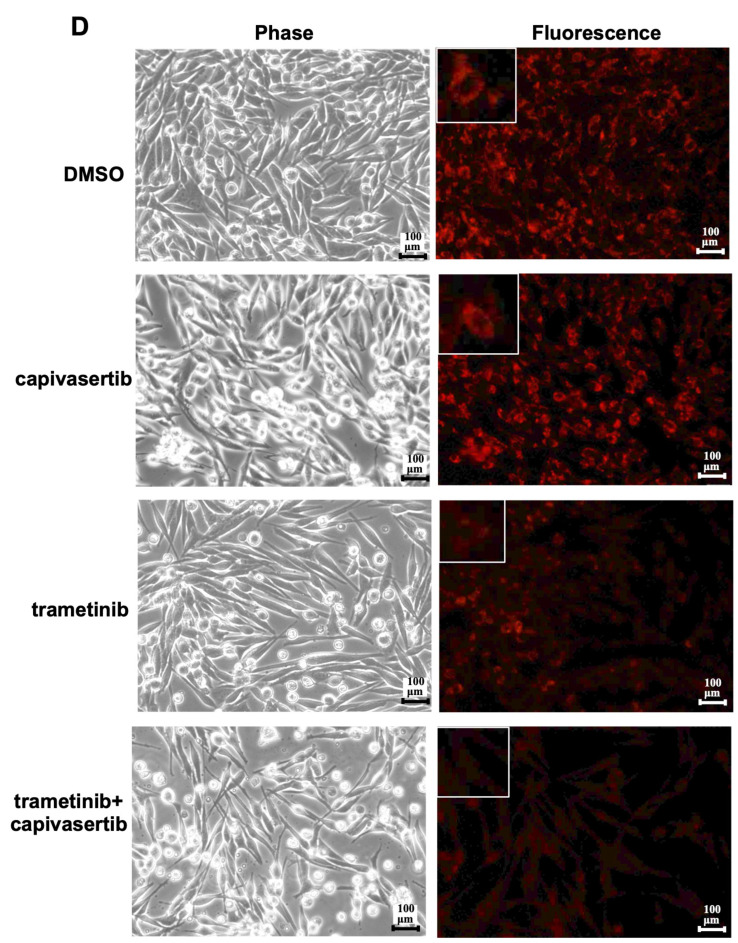
Capivasertib enhances apoptosis in trametinib-treated BAKP (**A**) and POT (**B**) melanoma cell lines. Cells were seeded in equal numbers in 6-well plates in triplicate and then treated with trametinib and/or capivasertib. After 48 h of treatment, cells were subjected to Annexin-APC/Sytox Blue apoptosis assays. The percentage of total apoptosis (the sum of early and late apoptosis in the lower-right and upper-right quadrants of the dot plots, respectively) were quantified via flow cytometric analysis. Results are means *±* SEM of three replicates of a representative experiment; results that were essentially the same were obtained in three independent experiments. *p* < 0.05 was considered significant; *, **, and **** represent *p* < 0.05, *p* < 0.01, and *p* < 0.0001, respectively. (**C**) Dot plot data used to generate the bar graphs in (**A**,**B**). (**D**) Representative phase contrast (**left** panel) and fluorescence (**right** panel) images of BAKP cells showing loss of mitochondrial membrane potential in BAKP cells treated with trametinib alone or in combination with capivasertib, but not in control cells or those incubated with capivasertib alone, indicating that apoptosis occurs through a mitochondrially mediated pathway. White-bordered squares show enlargement of select cells.

Based on CD133’s activation of an AKT/p-BAD survival pathway in trametinib-treated human melanoma stem cells as a mechanism for trametinib resistance, we next targeted the AKT pathway in BAKP and POT cells harboring NRAS^Q61K^ and NRAS^Q61R^ mutations, respectively. Cells were exposed for 48 h to trametinib and capivasertib, alone or in combination, and subjected to Annexin/SYTOX Blue flow cytometric assays. While the pan-AKT inhibitor capivasertib was not cytotoxic by itself, the addition of capivasertib to trametinib significantly enhanced the apoptotic activity induced by trametinib alone in BAKP (Figure 2A) and POT (Figure 2B) melanoma cell lines. The combination treatment was the most effective in inducing apoptosis in both cells lines. To determine whether this apoptosis was occurring through a mitochondrial-mediated pathway, BAKP cells were exposed for 48 h to capivasertib, trametinib alone, or a combination and then subjected to an assay to detect changes in mitochondrial membrane potential. The results show immunofluorescent images of the viable cells with red fluorescent mitochondria in control BAKP cells (exposed to the DMSO vehicle) and in those treated with capivasertib alone. In contrast, there was a slight decrease in mitochondrial staining in trametinib-treated cells, which decreased further in cells treated with the combination, thus indicating that apoptosis is indeed mediated via a mitochondrial-mediated mechanism (Figure 2D). This is consistent with our previous results, showing the proteolytic activation of caspase 9 [5]. 

To determine if ER stress is also involved in mediating trametinib+capivasertib-induced apoptosis, cell extracts were probed with markers of ER stress, including binding immunoglobulin protein (BiP)/glucose-regulated protein 78 (GRP78), and protein disulfide isomerase (PDI) [36]. The BiP and PDI levels were not markedly altered in the treated BAKP cells (Supplementary Figure S3).

### 3.4. Effects of CD133 Knockout or Induced CD133 Expression on Cell Viability of BAKP Cells Treated with MEKi Trametinib and AKT Inhibitor Capivasertib, Alone or in Combination

**Figure 3:**
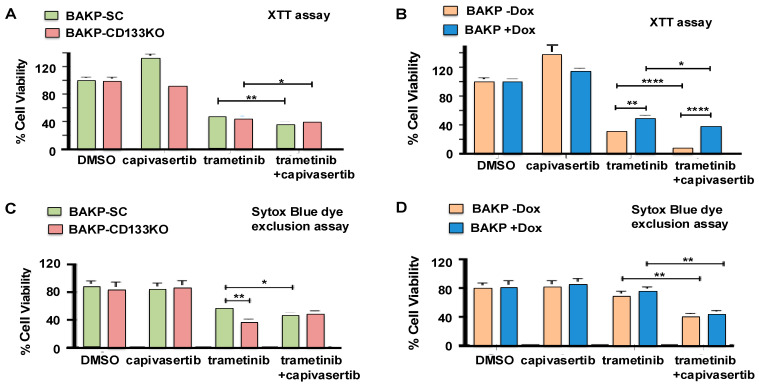
Effects of CD133 KO (**A**,**C**) or induced CD133 expression (**B**,**D**) on cell viability after treatment with trametinib and capivasertib, alone or in combination. Cells were plated in equal numbers in 6-well plates in triplicates and then treated for 48 h with trametinib and capivasertib, alone or in combination. The cells were then collected and subjected to XTT cell viability metabolic assays (**A**,**B**) and Sytox Blue dye exclusion assays (**C**,**D**). The percentage (%) of cell viability was quantified as described in Section 2. The results shown are the means *±* SEM of three replicates of a representative experiment; essentially the same results were obtained in three independent experiments. *p* < 0.05 was considered significant. *, **, and **** represent *p* < 0.05, *p* < 0.01, and *p* < 0.0001, respectively.

While capivasertib alone had no effect on cell viability, as assessed by the XTT metabolic assays, trametinib or the combination of trametinib + capivasertib significantly decreased the cell viability of the BAKP SC control or CD133 KO cells. The addition of capivasertib to trametinib further enhanced trametinib cytotoxicity in both cell lines (Figure 3A). On the other hand, Dox-induced CD133 expression markedly reversed trametinib cytotoxicity (Figure 3B), likely because CD133 activated an AKT/p-BAD survival pathway, increasing cell survival in trametinib-treated cells. Nevertheless, capivasertib appeared to overcome the effects of CD133, given that the addition of capivasertib to trametinib was able to further increase the cytotoxic effects of trametinib even in the CD133-overexpressing cells (Figure 3). Essentially the same results were obtained with SYTOX Blue dye exclusion assays, another type of cell viability assay (Figure 3C,D). The combination of trametinib + capivasertib therefore elicited significantly more cytotoxicity than trametinib alone, targeting even recalcitrant CD133-expressing melanoma stem cells.

### 3.5. Effects of CD133 KO on Apoptosis Induction in Response to Trametinib and Capivasertib in Single or Dual Combination as Assessed by Annexin Flow Cytometric Assays

**Figure 4:**
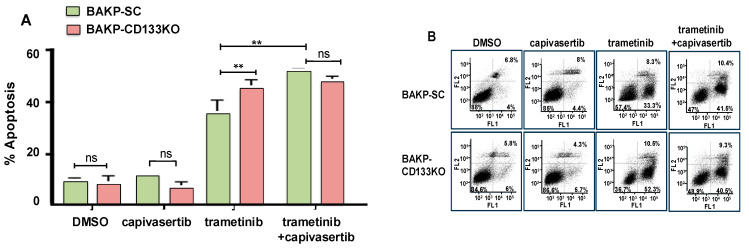
(**A**) Annexin flow cytometric assays to assess apoptosis induction after treatment with trametinib and capivasertib alone or in combination. (**B**) Dot plot of data shown in (**A**); FL1 and FL2 represent fluorescence channel 1 and fluorescence channel 2, respectively.Equal numbers of BAKP-CD133 KO and control BAKP-SC cells were seeded in 6-well plates in triplicate and then treated for 48 h with trametinib, capivasertib, or in combination. After 48 h, the cells were subjected to Annexin-APC/SYTOX Blue assays. The percentage of total apoptosis was quantified by flow cytometric analysis. The results shown are the mean *±* SEM of triplicates of a representative experiment; essentially the same results were obtained in three independent experiments. *p* < 0.05 was considered significant. ** represents *p* < 0.01; ns represents not significant.

We next determined whether trametinib and/or capivasertib exposure reduced cell viability by inducing apoptosis in CD133 KO or scrambled control cells. Cells were exposed for 48 h to trametinib, capivasertib, or combinations of the two drugs, followed by Annexin-APC/SYTOX Blue flow cytometric apoptosis assays (Figure 4). While capivasertib by itself did not induce apoptosis, CD133 depletion by KO significantly elevated apoptotic cell death in trametinib-treated melanoma cells. The addition of capivasertib to trametinib also markedly enhanced apoptosis in BAKP cells, mimicking the effects of CD133 KO and suggesting that CD133 activates an AKT survival pathway in response to trametinib treatment. In agreement with the XTT cell viability assays, the trametinib + capivasertib combination induced maximal apoptosis, to the same extent in both CD133-expressing SC and CD133 KO cells.

### 3.6. The Combination of Capivasertib and Trametinib Induces Maximal Cytotoxicity and Apoptosis, Which Cannot Be Reversed by Increased CD133 Expression in Dox-Inducible Cells

**Figure 5:**
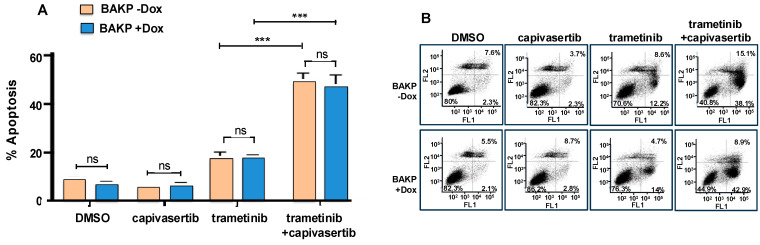
Capivasertib in combination with trametinib elicits a maximal apoptotic response in both uninduced and Dox-induced CD133-expressing cells, as assessed by annexin flow cytometric assays (**A**). CD133 expression slightly reverses this response. Cells were seeded in equal numbers in 6-well plates in triplicates, incubated for 24 h with 1 µg/mL Dox to induce CD133 expression, and then treated for 48 h (**A**) with trametinib and capivasertib, alone or in combination. Cells were collected after treatment and subjected to Annexin-APC apoptosis assays. The percentage (%) of total apoptosis was quantified by flow cytometric analysis. (**B**) Dot plot of data shown in **A**; FL1 and FL2 represent fluorescence channel 1 and fluorescence channel 2, respectively. The results shown are the means *±* SEM of three replicates of a representative experiment; essentially the same results were obtained in three independent experiments. *p* < 0.05 was considered significant. *** represents *p* < 0.001; ns represents not significant.

To further assess the effects of CD133 on the apoptotic response of melanoma cells exposed to single or combination treatment, Dox-inducible cells were incubated for 24 h with Dox to induce CD133 expression and then treated for 48 h with trametinib and capivasertib, alone or in combination, followed by Annexin flow cytometric apoptosis assays. Capivasertib in combination with trametinib elicited a maximal apoptotic response in both uninduced and Dox-induced CD133-expressing cells. Dox-induced CD133 expression only slightly reversed the response to the combination treatment (Figure 5). 

### 3.7. Capivasertib and Trametinib Function Synergistically in Melanoma Cells

**Figure 6:**
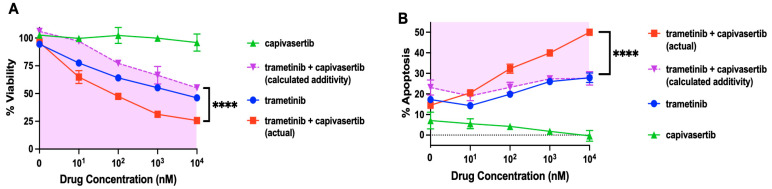
The combination of capivasertib plus trametinib synergistically reduces cell viability and induces apoptosis in melanoma cells. (**A**) Cells were seeded in equal numbers in 96-well plates in triplicate and then treated for 48 h with trametinib and capivasertib, alone or in combination (trametinib at variable concentrations and capivasertib at 1 µM). Cells were collected and subjected to XTT cell viability assays. (**B**) Cells were plated in a 6-well plate, exposed to the drugs alone or in combination as above, and then analyzed using flow cytometry after Annexin-APC/SYTOX Blue staining. (**A**,**B**) Bliss additivity was calculated and is shown as inverted triangles. Pink shading and unshaded areas represent synergy and antagonism, respectively. The results shown are the means *±* SEM of three replicates of a representative experiment; essentially the same results were obtained in three independent experiments. *p* < 0.05 was considered significant. **** represents *p* < 0.0001.

To determine whether the two drugs were functioning synergistically, dose–response experiments were performed with XTT cell viability and apoptosis assays in Dox-inducible melanoma cells treated with capivasertib and trametinib, alone or in combination (Figure 6). Remarkably, there was no loss of viability in cells exposed to any of the capivasertib doses tested. In contrast, cell viability was dose-dependently reduced with increasing concentrations of trametinib. Compared to trametinib alone, however, cell viability was further significantly decreased when the same doses of trametinib were used in combination with 1 µM capivasertib. The combination appeared to be functioning synergistically.

To assess whether the effect of the combination treatment was synergistic or additive, the Bliss independence testing method was used [33], whereby the additional response to trametinib was calculated as the fraction F_T_ x the remaining possible response (1 − F_C_). The total additive response of the mixture of the two drugs was calculated using the equation F_C_ + F_T_ (1 − F_C_) = F_C_ + F_T_ − F_C_ × F_T_. Since capivasertib exhibited no cytotoxicity by itself, F_C_=0, and the equation reduced to F_C_ + F_T_ (1 − F_C_) = F_C_ + F_T_ − F_C_ × F_T._= 0 + F_T_ – 0 × F_T_ = F_T;_, which would have corresponded to the calculated value had the cytotoxic effect been additive. Given that cell viability in response to the combination treatment was significantly lower than the calculated Bliss additive response, the combination was therefore synergistic (Figure 6A, pink shaded area). Trametinib concentrations of 10, 10^2^, 10^3^, and 10^4^ nM therefore induced a synergistically greater decrease in cell viability in the presence of capivasertib. 

The levels of apoptosis were likewise examined in dose–response experiments using Annexin-APC/SYTOX Blue flow cytometric assays. Consistent with the results of the XTT cell viability assays, 10^2^, 10^3^, or 10^4^ nM trametinib in combination with 1 µM capivasertib also synergistically increased apoptotic cell death in melanoma cells (Figure 6B). The extent of apoptosis elicited by the combination treatment was found to be significantly greater than the calculated Bliss additive response, thus confirming that the combination was synergistic.

### 3.8. Long-Term Cell Survival Is Decreased by Trametinib or the Combination of Capivasertib + Trametinib, an Effect Which Is Reversed by Dox-Induced CD133 Expression

**Figure 7:**
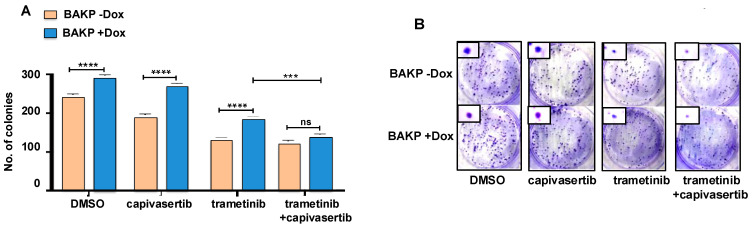
(**A**) Long-term cell survival (clonogenic) assays reveal that treatments reduce colony formation in BAKP cells, with the combination of trametinib + capivasertib decreasing colony formation to the largest extent. Dox-inducible BAKP cells were incubated for 24 h with Dox, and then exposed to trametinib and capivasertib by themselves or in combination. Then, 48 h after treatment, the cells were replated, allowed to grow for 12 days, fixed, and stained, and the colonies of cells that survived treatment were counted. The results shown are the means *±* SEM of three replicates of a representative experiment; essentially the same results were obtained in three independent experiments. *p* < 0.05 was considered significant. *** and **** represent *p* < 0.001 and *p* < 0.0001, respectively; ns represents not significant. (**B**) Images of representative 10 cm-plates with stained colonies reveal that the treatments reduced both colony counts and colony sizes. The insets show representative colony sizes.

Clonogenic assays were then used to determine the long-term ability of cells to survive and self-renew following treatment. Dox-inducible BAKP cells were incubated for 24 h without or with Dox to induce CD133 expression, and they were then treated for 48 h with trametinib and capivasertib alone or in combination. Following treatment, the cells were plated at clonal density, allowed to grow for 12 days, and the cell colonies were counted. Consistent with the cell viability and apoptosis assays, while capivasertib alone had no effect on the number of colonies, trametinib-treated cells exhibited significantly lower colony counts, indicative of diminished cell survival, compared to the DMSO controls (Figure 7). However, the combination of trametinib + capivasertib was the most effective at decreasing long-term cell survival, as evident from the significantly fewer colony counts compared to trametinib or capivasertib alone. Importantly, the induction of CD133 expression in +Dox cells partially but significantly prevented the decrease in colony number in cells treated with trametinib or capivasertib alone, but not their combination, indicating that CD133 only increased long-term survival in single-agent-treated cells. For all treatment groups, except for the combination, CD133 expression in +Dox cells increased colony formation, partially reversing the drug’s effects.

### 3.9. The Combination of Capivasertib + Trametinib Inhibits the Phosphorylation of BAD and GSK-3β and Increases Caspase-3 Activation in Melanoma Cells

**Figure 8:**
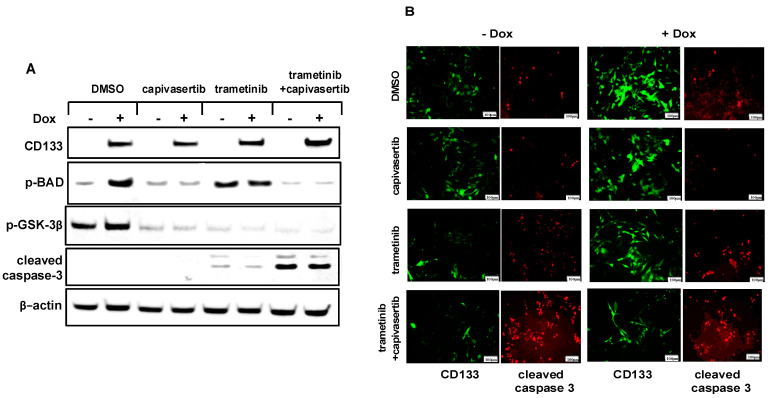
(**A**) The immunoblot analysis reveals the effective inhibition of phosphorylation of AKT substrates (p-BAD and p-GSK-3β) by capivasertib by itself or in combination with trametinib. BAKP cells were incubated for 24 h with Dox and then exposed to trametinib or capivasertib alone or in combination with trametinib. Cell lysates were then subjected to immunoblot analysis with antibodies specific for CD133, phospho-BAD, p-GSK-3β, and cleaved caspase-3. Anti-β-actin was used for the confirmation of equal loading. (**B**) The indirect immunofluorescent analysis with antibodies to CD133 or the cleaved active form of caspase-3 reveals that treatment with a combination of trametinib (100 nM) + capivasertib (1 μM), but not either drug alone, markedly increases apoptotic caspase-3 activation in BAKP cells.

To determine whether capivasertib inhibited the cell survival pathways activated by CD133, the cells were induced with Dox and then treated with trametinib, capivasertib, or a combination of the two. Immunoblot analysis to examine the activation of downstream proteins revealed that capivasertib markedly suppressed the phosphorylation of GSK-3β, a canonical marker for AKT activation, alongside another AKT substrate, BAD (Figure 8). The phosphorylation of BAD was suppressed in all cells treated with capivasertib, regardless of whether trametinib was present. The level of the cleaved activated form of caspase-3, a marker for apoptosis, was only slightly increased by trametinib alone, but it was dramatically augmented by the combination therapy. Thus, capivasertib appeared to contribute to cell death in combination therapy in part by suppressing BAD phosphorylation via AKT, which converted the pro-apoptotic BAD to a pro-survival p-BAD protein. CD133 was unable to block caspase-3 activation in +Dox cells, consistent with its role upstream of AKT. In agreement with the immunoblot analysis, more cells exposed to the combination therapy showed markedly increased levels of proteolytically cleaved activated caspase-3 compared to either drug alone, as shown by the immunoblot (Figure 8A) or immunofluorescent analysis (Figure 8B), regardless of CD133 induction.

### 3.10. Capivasertib and Trametinib Function Synergistically in Melanoma Xenografts

**Figure 9:**
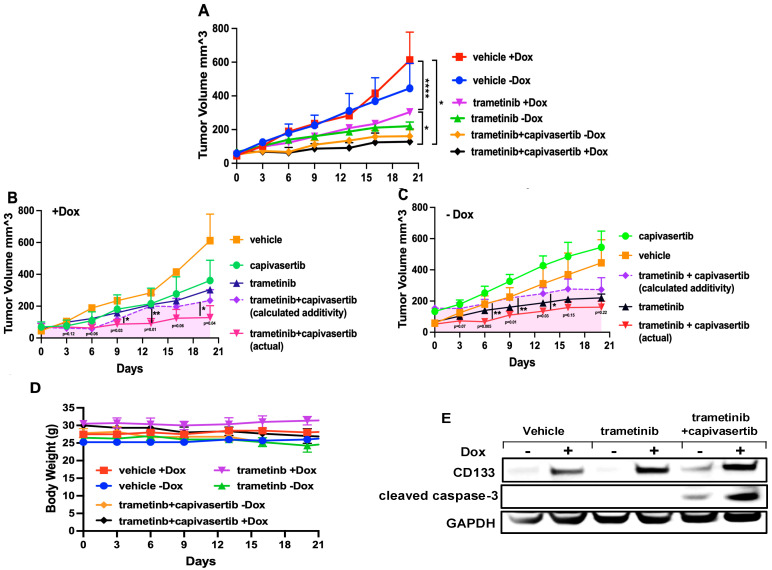
CD133 increases tumor growth, which is suppressed by the trametinib + capivasertib treatment in vivo. BAKP-inducible cells were used to induce subcutaneous tumors. (**A**) Tumor volumes in treated vs. vehicle control mice (+/− Dox). Bliss additivity was calculated in +Dox mice (**B**) or −Dox mice (**C**), and is shown as a dashed purple line, while the pink-shaded areas show regions where combination treatment demonstrates synergy and improvement over trametinib alone. (**D**) No effects on the body mass of mice over the treatment period were observed. (**E**) The immunoblot analysis of tumor lysates from xenografted mice shows apoptotic caspase-3 cleavage in the combination treatment only. The results shown are the means *±* SEM of three replicates of a representative experiment; essentially the same results were obtained in three independent experiments. *p* < 0.05 was considered significant. *, **, and **** represent *p* < 0.05, *p* < 0.01, and *p* < 0.0001, respectively.

Since the combination treatment was effective at inducing apoptosis in cultured melanoma cells, we tested its efficacy in vivo in murine xenografts. To help monitor the fate of tumors, BAKP Dox-inducible CD133 cells were injected subcutaneously into the hind flanks of nude mice. Half of the mice that were xenografted with BAKP Dox-inducible cells were fed with Dox-supplemented feed to induce CD133 expression. Tumor growth in mice was monitored with calipers using the following formula: tumor volume = 1/2 length × width^2^. When the tumor volumes reached 100 mm^3^, drug treatment was performed by oral gavage with a vehicle emulsion control, trametinib (3 mg/kg/qd; daily), capivasertib (100 mg/kg/qod; every other day), either alone or in combination. Drug efficacy was monitored daily, as well as the adverse effects (loss of weight), and necropsy was performed upon the sacrifice of each mouse, due to either tumor burden or the termination of the experiment. Daily monitoring of each of the treatment groups yielded tumor growth curves that revealed markedly different rates of tumor growth (Figure 9A). First, Dox-induced CD133 in BAKP vehicle control tumors formed rapidly growing tumors that increased in area over the 21-day course of the experiment. The second fastest rate of tumor growth was observed in the uninduced BAKP cells treated with vehicle alone. Mice receiving oral trametinib had tumors that grew at a slower rate than vehicle-fed mice. Trametinib + capivasertib-treated mice had tumors that grew substantially slower than the trametinib group. In fact, the combination drug treatment essentially stopped the growth of tumors, regardless of CD133 status. Thus, the combination therapy appears to overcome the effects of induced CD133. In both the presence (Figure 9B) and absence of Dox (Figure 9C), a synergistic effect of the combination therapy was observed (CI values < 1 in Supplementary Tables S3 and S4).

Necropsy did not reveal any abnormalities in the animals in any of the groups, nor was there any reduction in the weights of the animals (Figure 9D). The immunoblot analysis of the tumor extracts revealed that CD133 was induced in Dox-fed animals. Further, cleaved activated caspase-3 was increased in animals treated with the combination of capivasertib + trametinib, consistent with the smaller tumor size in this group (Figure 9E).

## 4. Discussion

**Figure 10:**
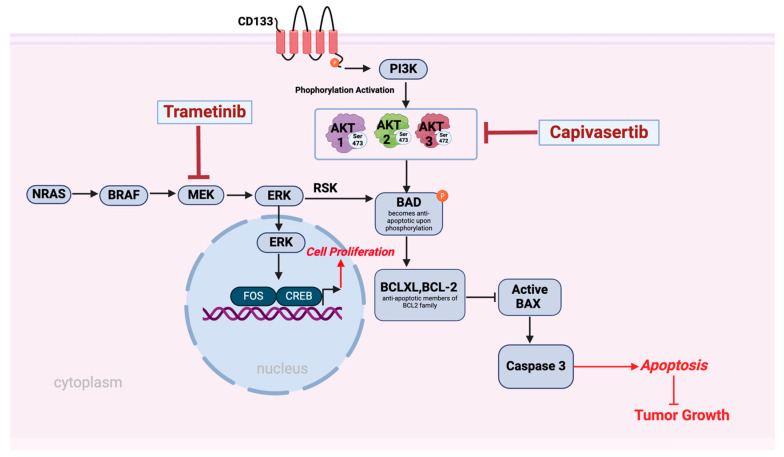
PI3K/AKT/Bcl2 and MAPK family pro-survival signaling pathway in CD133+ melanoma stem cells (MICs) and possible therapeutic targets.

In this study, we showed that CD133 likely activates an AKT-dependent survival pathway (summarized schematically in Figure 10) that renders melanoma cells resistant to the MEK inhibitor trametinib. CD133 phosphorylation can promote its binding to the phosphoinositide 3-kinase (PI3K) p85 subunit, which phosphorylates and activates AKT [37,38]. We also performed co-immunoprecipitation experiments confirming the binding of the p85 subunit of PI3K to CD133 (Supplementary Figure S4). We consistently showed that the induction of CD133 expression results in the phosphorylation and activation of AKT and in the phosphorylation of its downstream substrates, such as BAD and GSK-3β. Thus, this activation may occur through direct binding of p85 PI3K to the cytoplasmic domain of CD133. PI3K/AKT may also be indirectly activated through the CD133-mediated upregulation of amphiregulin and the EGFR pathway [39]. In the current study, we demonstrated that we can leverage this finding using capivasertib in combination with trametinib to enhance apoptosis in cell cultures and block tumor growth in vivo. Capivasertib was chosen because it is a potent pan-caspase inhibitor that recently received FDA approval for breast cancer in combination with fulvestrant [40]. Capivasertib alone does not suppress melanoma cell growth in culture (Figure 2, Figure 3, Figure 4 and Figure 5). This is in agreement with previous studies in which other AKT inhibitors did not show any effect on cell proliferation or survival when used as a monotherapy [41]. The advantage of using inhibitors of MEK and AKT is that their combined effects may provide a larger therapeutic window. Consistent with this idea, our current in vivo results with xenografted mice suggest that doses sufficient for tumor growth suppression do not appear to induce any significant adverse effects, as judged by weight maintenance, and the absence of any visible pathology, as determined by necropsy (Figure 9D). We are currently performing knockout experiments on each of the three AKT genes to determine which, if any, paralogue is most important for melanoma initiation, growth, chemoresistance, and metastasis. This will allow more selective AKT paralogue-specific inhibitors to be developed for combination therapy that is even more selective to tumors, hopefully with fewer side effects. A recent study showed that the genetic silencing of all AKT paralogs induced melanoma cell death [41], although, in that case, an mTOR pathway downstream of AKT was implicated, as opposed to our findings, which implicated the AKT depletion-mediated dephosphorylation of BAD, which increased BAD-mediated apoptosis, given that navitoclax, a Bcl2 inhibitor, could substitute for CD133 or AKT depletion [5]. Further, the role of CD133 was unknown in this previous study.

The melanoma cells in this study were derived from human NRAS-mutant tumors, which are difficult to treat with targeted kinase inhibitors compared to BRAF-mutant tumors. As a result, most clinical trials do not focus on an NRAS-mutant cohort arm, and no study has examined melanoma stem cells. One study that had a small NRAS-mutant cohort arm (NCT02465060; *n* = 10) did not show clinical benefit from a combination of an AKT inhibitor with an MAPK inhibitor [42]. However, the clinical trial utilized a different AKT inhibitor, uprosertib (*GSK2141795*), a pan-Akt inhibitor with IC50 values of 180/328/38 nM for Akt1/Akt2/Akt3, respectively, which are much higher than those for capivasertib with an IC50 of < 10 nm for the three AKT paralogue isoforms. Our studies are in excellent agreement with a previous study [43], in which the authors showed that Buparlisib (BKM120), an oral pan-class I PI3K inhibitor targeting all isoforms of PI3K [44], worked in combination with trametinib to induce apoptosis and suppressed tumor growth in nude mice. In this case, different BRAF-mutant melanoma cell lines were again used, as opposed to our NRAS-mutant melanoma-based study. There have also been a small number of clinical trials in which PI3K inhibitors have been used in combination with vemurafinib in the treatment of melanoma [45,46].

In the current study, we showed a role for CD133 in increased melanoma cell survival after trametinib treatment, which can be ameliorated by capivasertib-mediated AKT inhibition in vitro and in vivo. We also demonstrated in another study that CD133 may be activating EGFR via amphiregulin [45,46], which, in turn, can further upregulate phosphorylation and the activation of AKT. Accordingly, to target CD133, several aptamers have been developed and used to deliver payloads to CD133+ cells [47]. An aptamer to a specific AKT inhibitor payload may also increase specificity.

## 5. Conclusions

In conclusion, our results together show that capivasertib works synergistically with trametinib to inhibit the growth of drug-resistant NRAS-mutant melanoma CSCs, both in vitro and in vivo. Targeting the CD133/AKT/survival pathways with capivasertib, together with the MEKi trametinib, underscores the potential for combinatorial therapies for the development of more effective treatments for melanoma patients with difficult-to-treat NRAS mutations. We have shown the feasibility of using two FDA-approved drugs for the effective treatment of human melanoma in vivo, with no detectable adverse outcomes. The simultaneous inhibition of MAPK, AKT, and/or CD133 and focusing on specific AKT paralogues may be viable strategies for the treatment of recalcitrant NRAS-mutant melanoma.

## Data Availability

The original contributions presented in this study are included in the article/Supplementary Materials; further inquiries can be directed to the corresponding author.

## Supplementary Materials

The following supporting information can be downloaded at https://www.mdpi.com/article/10.3390/cells14040248/s1: Figure S1: Full-length gels of immunoblots used in the figures. Figure S2: Time Course Experiment (of CD133 expression and caspase 3 activation by cleavage in Dox-inducible BAKP cells treated for 8 h, 24 h, or 48 h with vehicle (DMSO), capivasertib, trametinib, or the combination of capivasertib + trametinib. The 48-hour treatment with the combination of capivasertib + trametinib induced the highest activation of caspase 3, an apoptotic marker. Figure S3: Apoptosis induced in BAKP melanoma cells after treatment with trametinib, alone or in combination with capivasertib, is not associated with increased ER stress. Cells were treated with Dox for 24 h to induce CD133 expression, then incubated for 48 h with capivasertib, trametinib, or the combination of the two. Cells were then collected and total cell lysates were subjected to immunoblot analysis with antibodies BiP (GRP78) and PDI, markers of ER stress signaling. Membranes were reprobed with antibodies to β-actin for confirmation of equal protein loading. Figure S4: Immunoprecipitation experiment verifying the binding of the p85 subunit of PI3K to CD133. BAKP cells were induced to express CD133 by incubation for 24 h with Dox, and cell lysates were extracted and subjected to immunoprecipitation with anti-CD133 bound to beads. Beads were washed, centrifuged down, and then subjected to SDS-PAGE and immunoblot analysis with anti-p85 subunit of PI3K. Table S1: CI values for Figure 6A XTT Cell Viability Assays. Table S2: CI values for Figure 6B Annexin Apoptosis Assays. Table S3: CI values for Figure 9B Tumor Growth +Dox. Table S4: CI values for Figure 9B Tumor Growth-Dox.
