# Correction: Alobaidi et al. Combinational Inhibition of MEK and AKT Synergistically Induces Melanoma Stem Cell Apoptosis and Blocks NRAS Tumor Growth. *Cells* 2025, *14*, 248

**DOI:** 10.3390/cells15080704

**Published:** 2026-04-16

**Authors:** Ryyan Alobaidi, Nusrat Islam, Toni Olkey, Yogameenakshi Haribabu, Mathew Shamo, Peter Sykora, Cynthia M. Simbulan-Rosenthal, Dean S. Rosenthal

**Affiliations:** 1 Department of Biochemistry and Molecular & Cellular Biology, Georgetown University School of Medicine, Washington, DC 20057, USA; raa125@georgetown.edu (R.A.); ni98@georgetown.edu (N.I.); tmo49@georgetown.edu (T.O.); yh577@georgetown.edu (Y.H.); mms420@georgetown.edu (M.S.); simbulac@georgetown.edu (C.M.S.-R.); 2 Department of Pathology, King Saud University College of Medicine, Riyadh 11461, Saudi Arabia; 3 Amelia Technologies, LLC, Washington, DC 20001, USA; peters@ameliatechnologies.com

## Error in Figure

In the original publication [1], there were two mistakes in Figure 2C as published. After the dot plot data used in Figures 2C and 5B were re-examined, the dot plot results used in Figure 5B for trametinib (T) and trametinib+capivasertib (T+C)-treated+Dox BAKP cells were determined to have been inadvertently duplicated and used for the Figure 2C dot plot for T and T+C-treated-Dox BAKP cells. Figure 5B is correct; however, the duplicated dot plot data in Figure 2C need to be replaced with the correct dot plot data for T and T+C-treated-Dox BAKP cells. The corrected Figure 2 appears below. 

The authors state that the scientific conclusions of this paper are unaffected. This correction was approved by the Academic Editor. The original publication has also been updated.

## Figures and Tables

**Figure 2:**
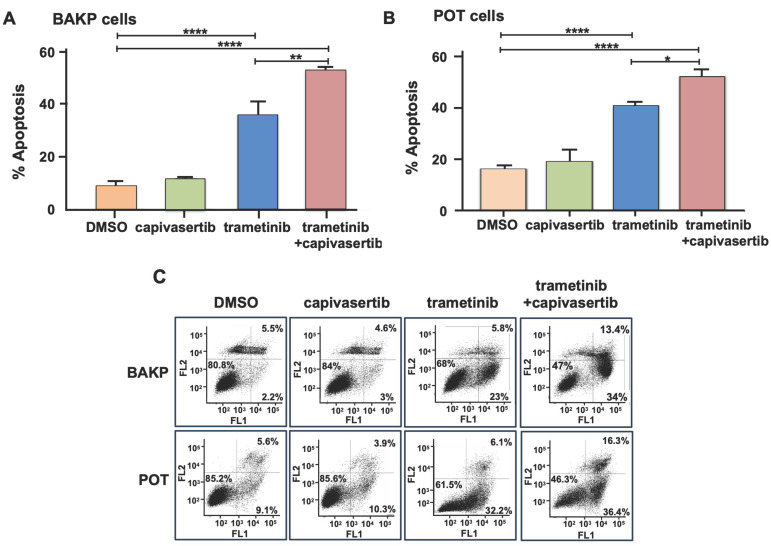

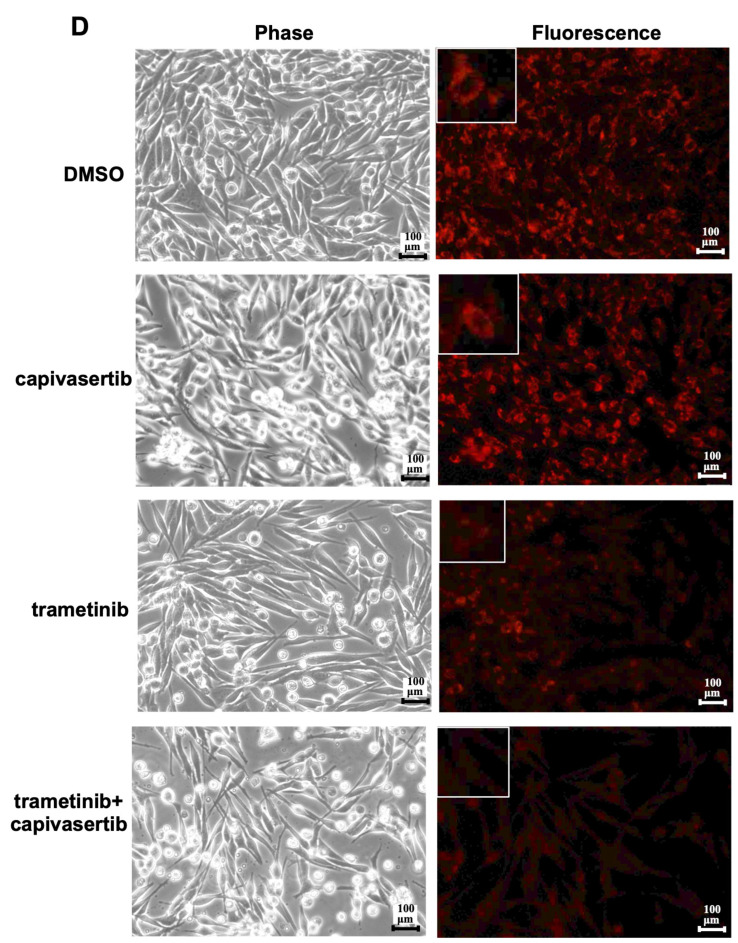
Capivasertib enhances apoptosis in trametinib-treated BAKP (**A**) and POT (**B**) melanoma cell lines. Cells were seeded in equal numbers in 6-well plates in triplicate and then treated with trametinib and/or capivasertib. After 48 h of treatment, cells were subjected to Annexin-APC/Sytox Blue apoptosis assays. The percentage of total apoptosis (the sum of early and late apoptosis in the lower-right and upper-right quadrants of the dot plots, respectively) were quantified via flow cytometric analysis. Results are means *±* SEM of three replicates of a representative experiment; results that were essentially the same were obtained in three independent experiments. *p* < 0.05 was considered significant; *, **, and **** represent *p* < 0.05, *p* < 0.01, and *p* < 0.0001, respectively. (**C**) Dot plot data used to generate the bar graphs in (**A**,**B**). (**D**) Representative phase contrast (**left** panel) and fluorescence (**right** panel) images of BAKP cells showing loss of mitochondrial membrane potential in BAKP cells treated with trametinib alone or in combination with capivasertib, but not in control cells or those incubated with capivasertib alone, indicating that apoptosis occurs through a mitochondrially mediated pathway. White-bordered squares show enlargement of select cells.

## References

[1] Alobaidi R., Islam N., Olkey T., Haribabu Y., Shamo M., Sykora P., Simbulan-Rosenthal C.M., Rosenthal D.S. (2025). Combinational Inhibition of MEK and AKT Synergistically Induces Melanoma Stem Cell Apoptosis and Blocks NRAS Tumor Growth. Cells.

