# Supplementary material for: Combinational Inhibition of MEK and AKT Synergistically Induces Melanoma Stem Cell Apoptosis and Blocks NRAS Tumor Growth
Source: Cells. 2025 Feb 10;14(4):248. doi: 10.3390/cells14040248 (PMC11852824; doi:10.3390/cells14040248)
Supplement: Supplementary file 1 [file cells-14-00248-s001.zip › cells-3426887-supplementary.pdf]

Supplementary Figures
Figure S1
Full Gel Images

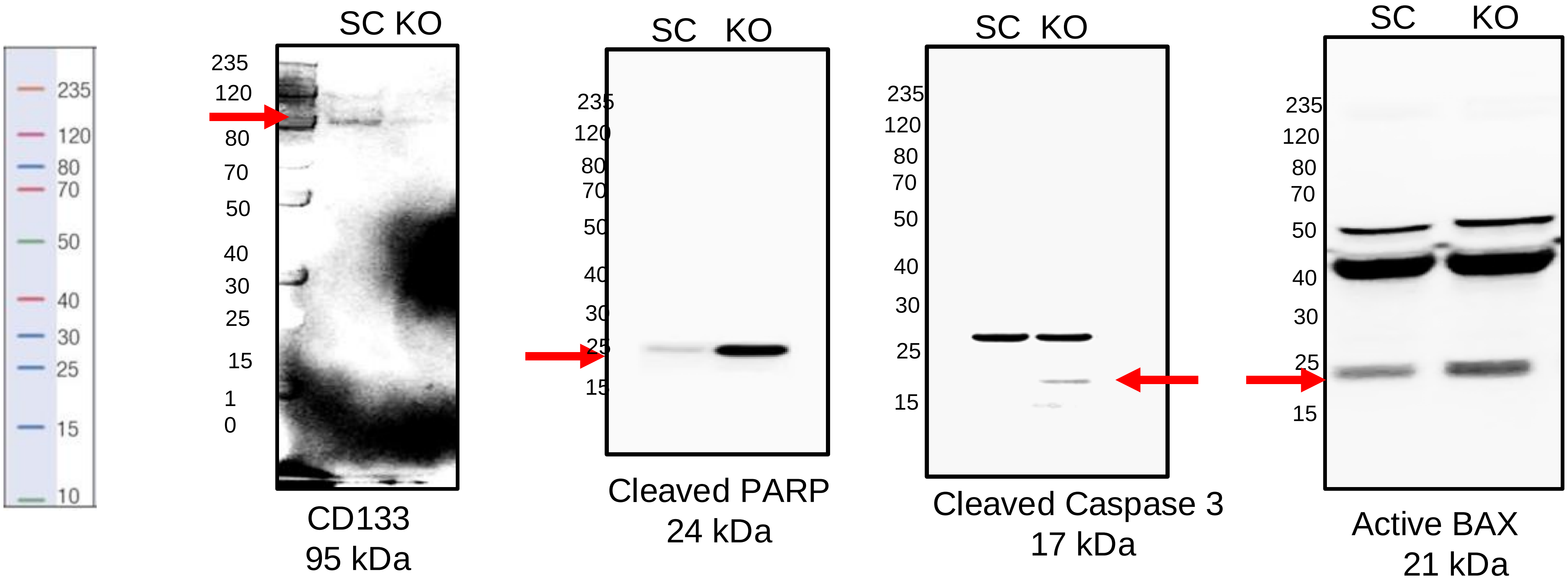

235
120
80
70
50
40
30
25
15
10
SC KO
235
120
80
70
50
40
30
25
15
1
0
CD133
95 kDa
SC KO
235
120
80
70
50
40
30
25
15
Cleaved PARP
24 kDa
SC KO
235
120
80
70
50
40
30
25
15
Cleaved Caspase 3
17 kDa
SC KO
235
120
80
70
50
40
30
25
15
Active BAX
21 kDa

Figure S1A

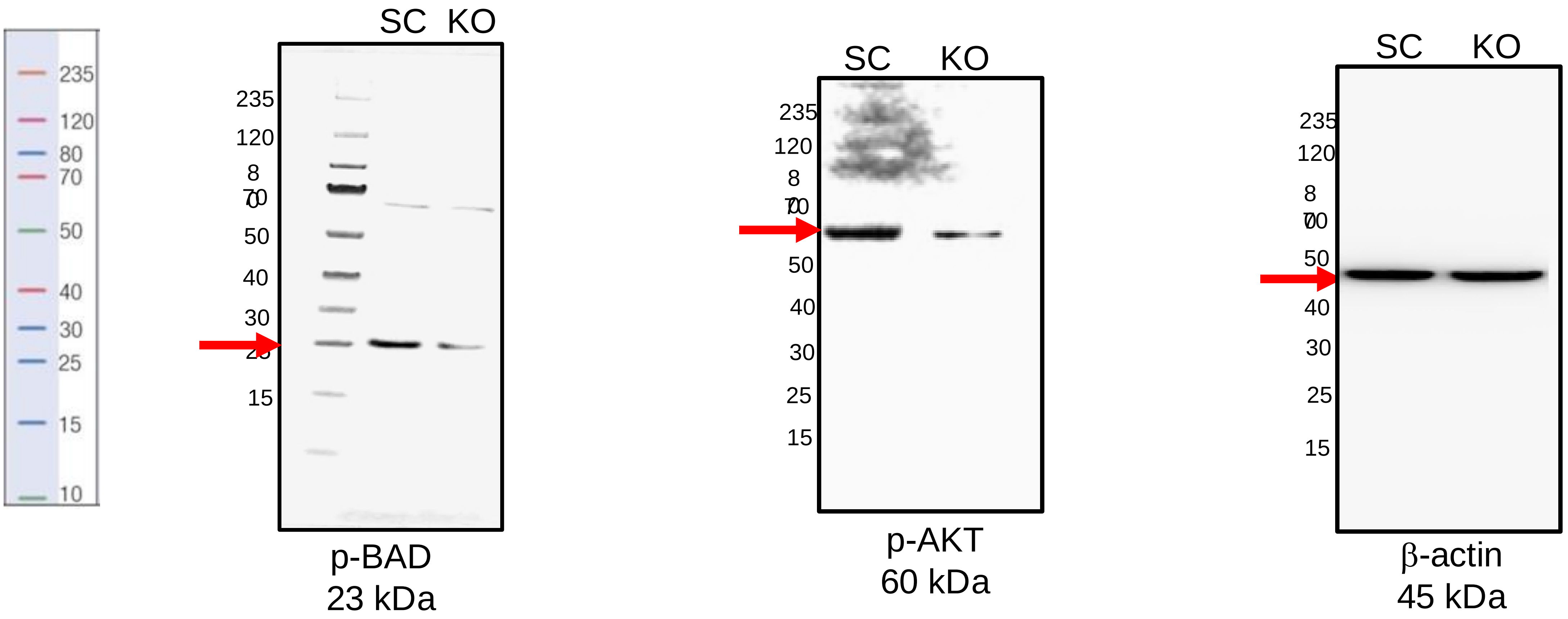
235
120
80
70
50
40
30
25
15
10
SC KO
p-BAD
23 kDa
SC KO
p-AKT
60 kDa
SC KO
β-actin
45 kDa

Figure S1A

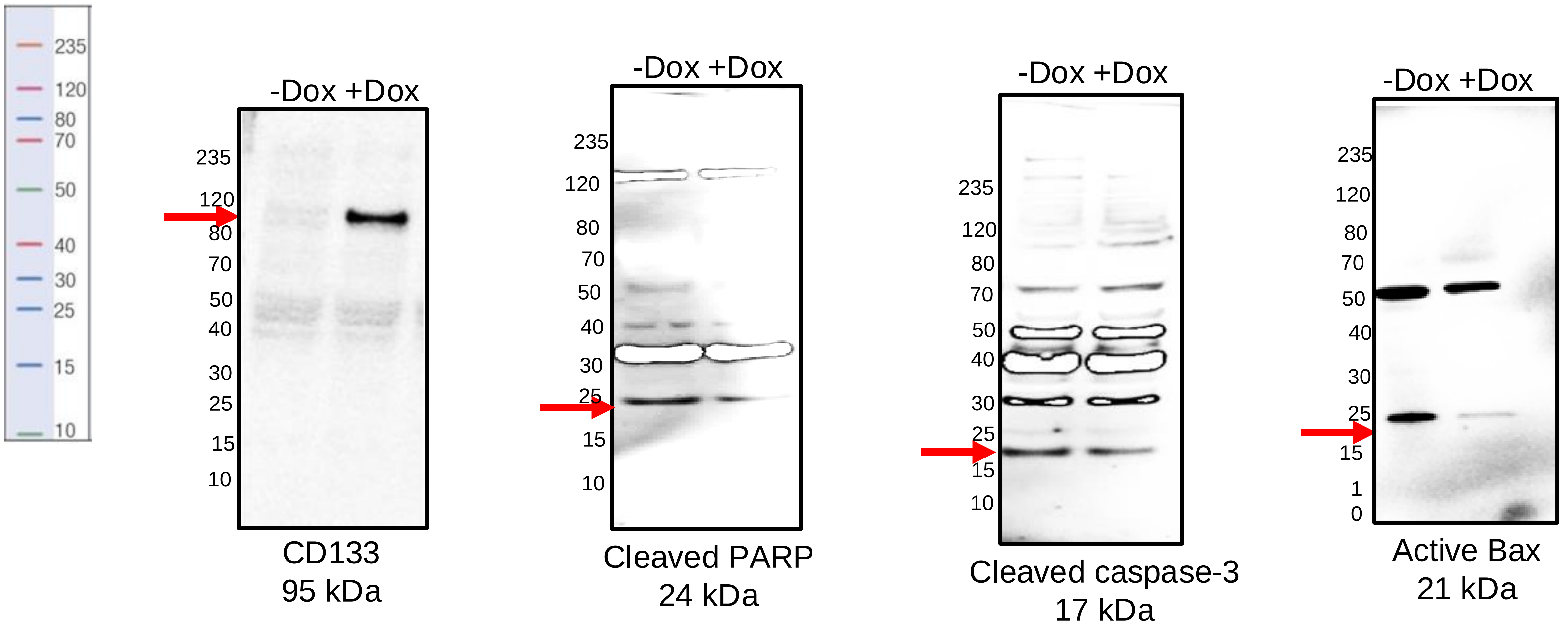
235
120
80
70
50
40
30
25
15
10
-Dox +Dox
CD133
95 kDa
Cleaved PARP
24 kDa
Cleaved caspase-3
17 kDa
Active Bax
21 kDa

Figure S1B

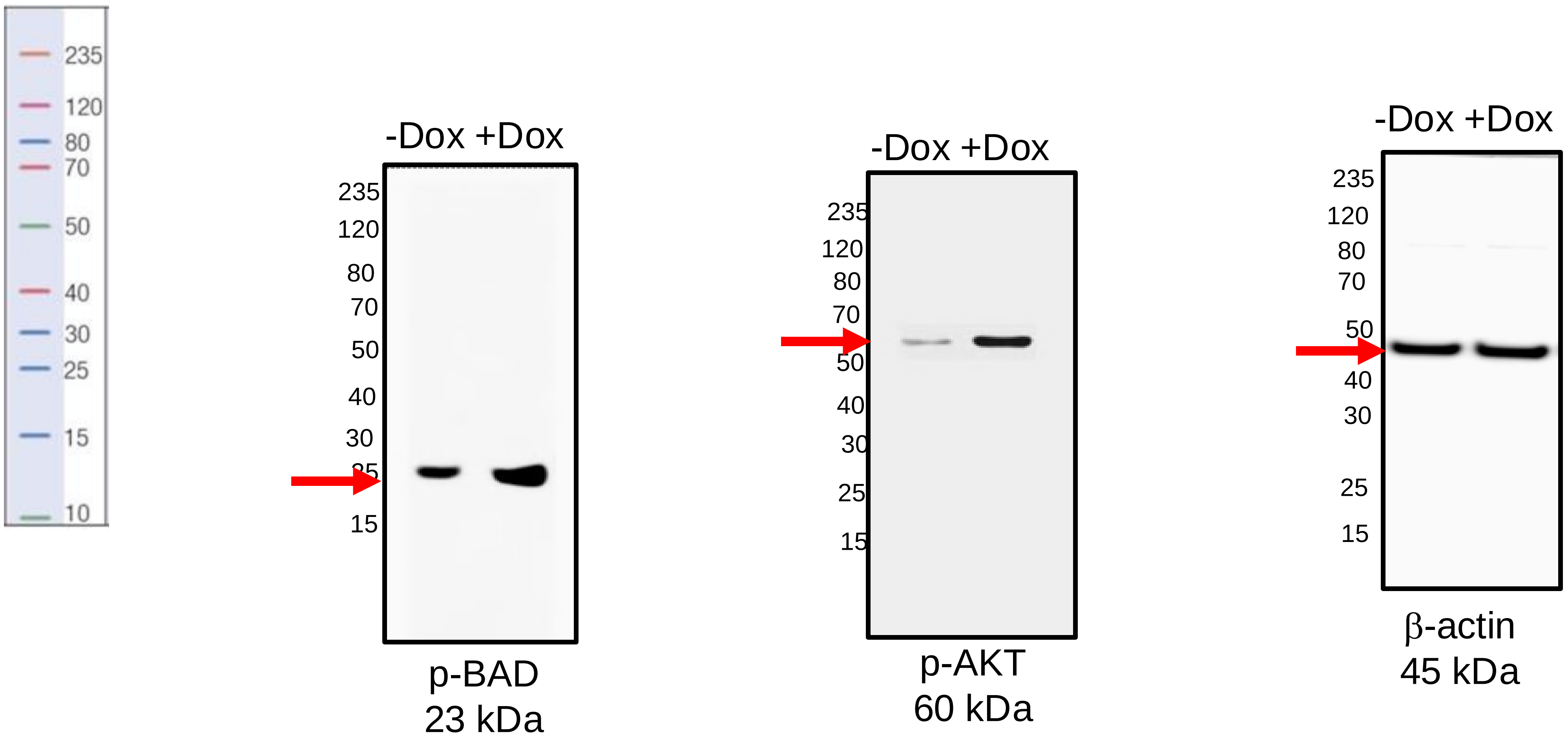
235
120
80
70
50
40
30
25
15
10
-Dox +Dox
235
120
80
70
50
40
30
25
15
p-BAD
23 kDa
-Dox +Dox
235
120
80
70
50
40
30
25
15
p-AKT
60 kDa
-Dox +Dox
235
120
80
70
50
40
30
25
15
β-actin
45 kDa

Figure S1B

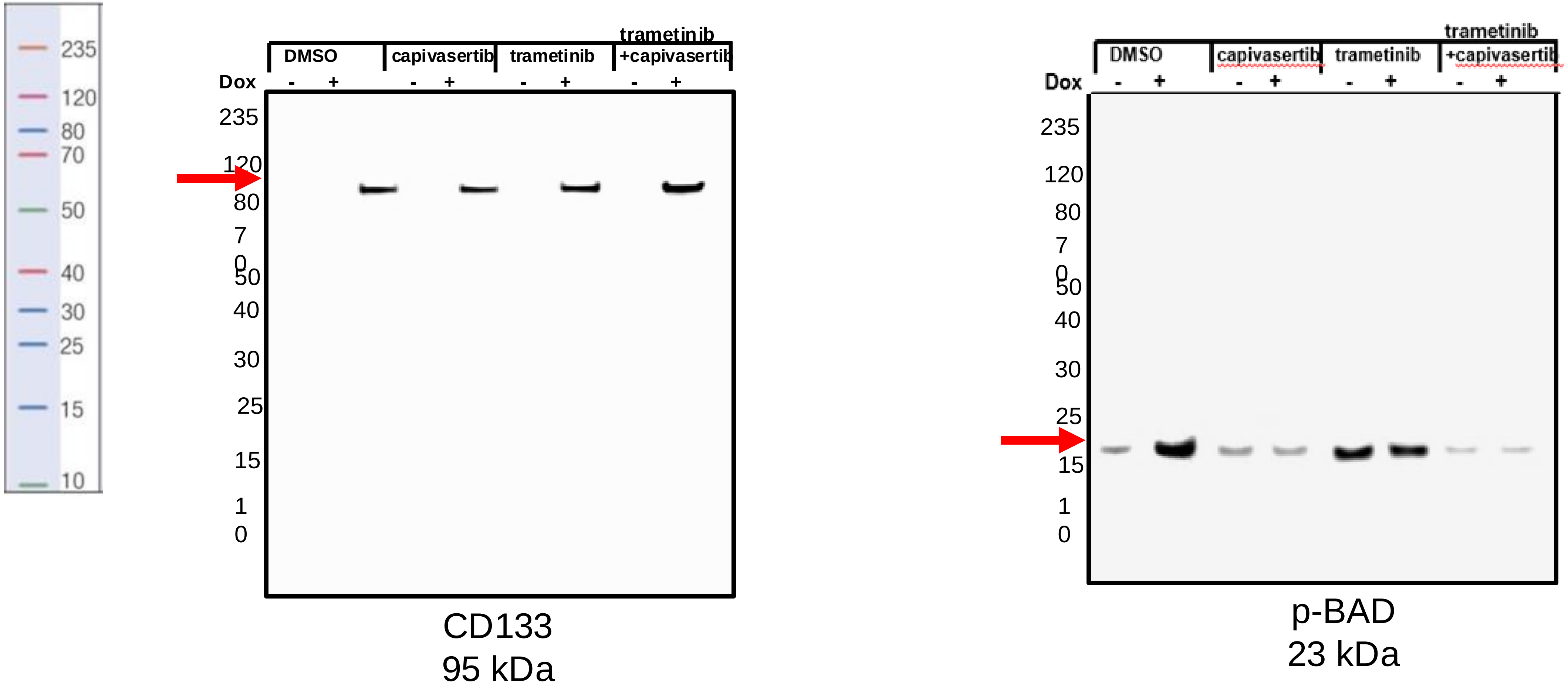

235
120
80
70
50
40
30
25
15
10
trametinib
DMSO
capivasertib
trametinib
+capivasertib
Dox - + - + - + - +
235
120
80
7
0
50
40
30
25
15
1
0
CD133
95 kDa
trametinib
DMSO
capivasertib
trametinib
+capivasertib
Dox - + - + - + - +
235
120
80
7
0
50
40
30
25
15
1
0
p-BAD
23 kDa

Figure S8A

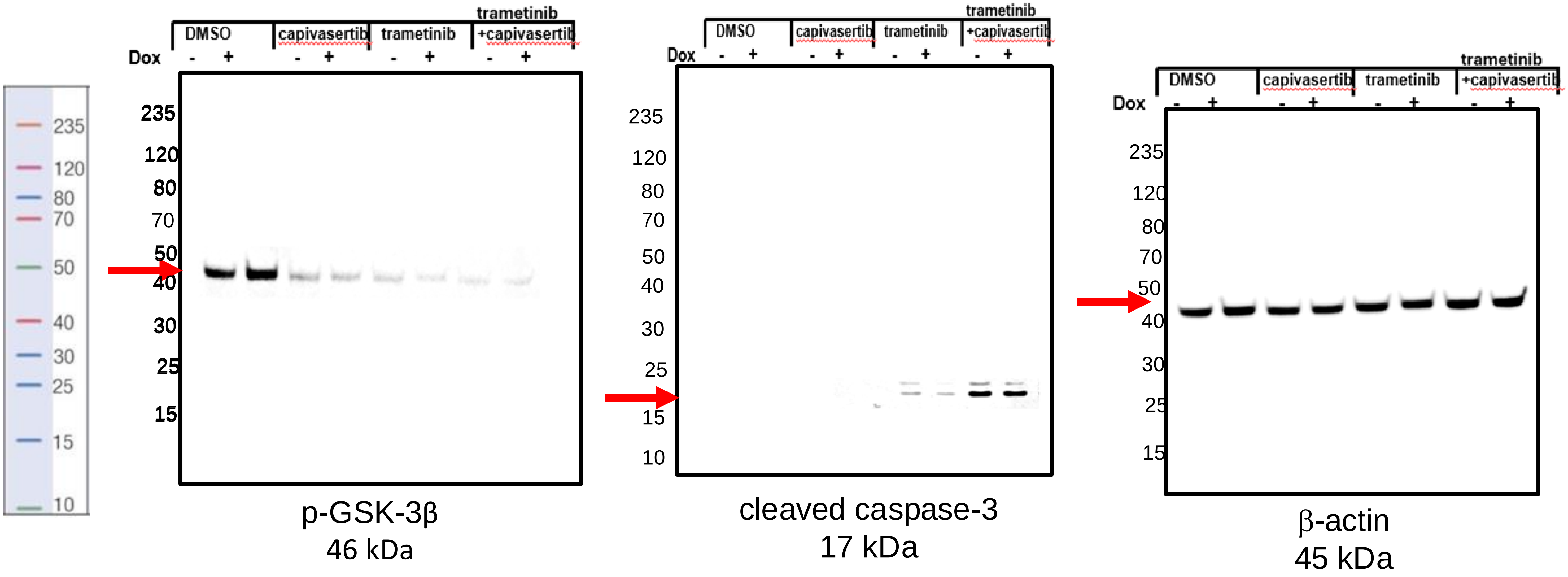

235
120
80
70
50
40
30
25
15
10
trametinib
DMSO
capivasertib
trametinib
+capivasertib
Dox
- + - + - + - +
p-GSK-3β
46 kDa
cleaved caspase-3
17 kDa
β-actin
45 kDa

Figure S8A

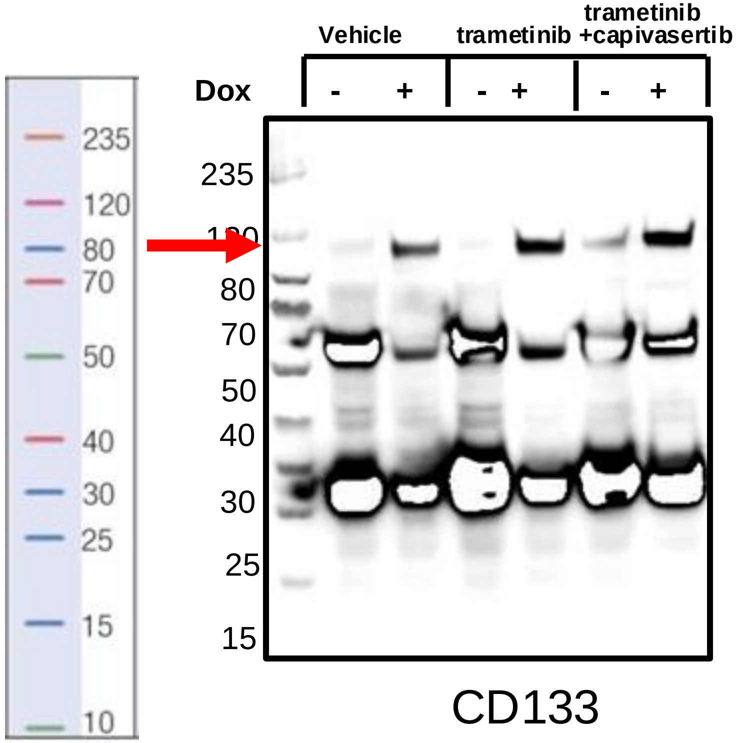

CD133
95 kDa

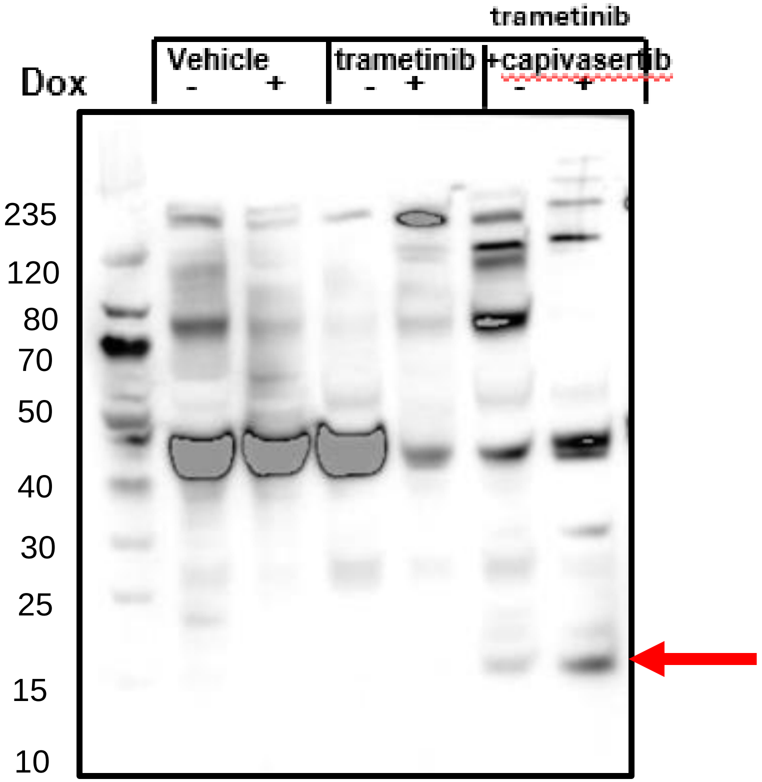

Cleaved Caspase 3
17 kDa

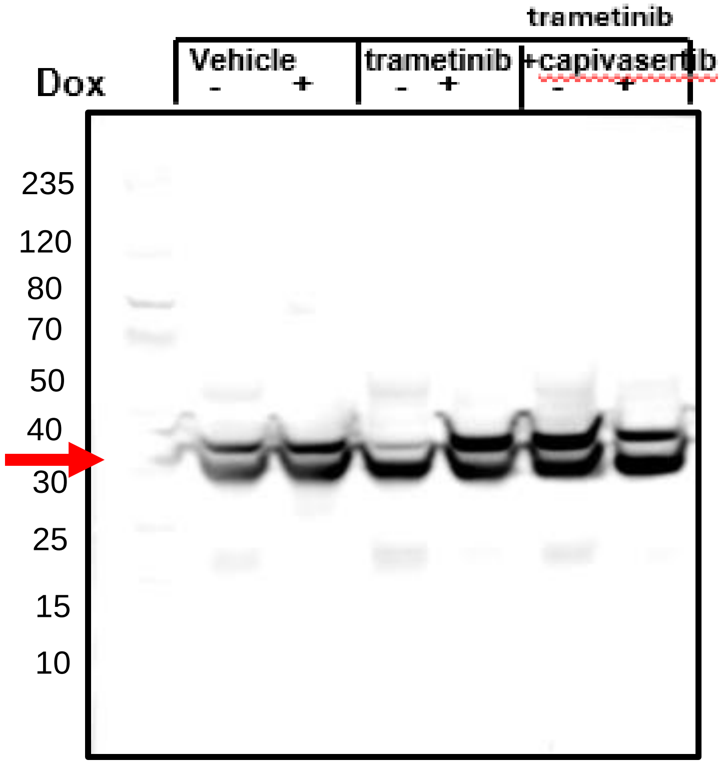

GAPDH
37 kDa

Figure S9E

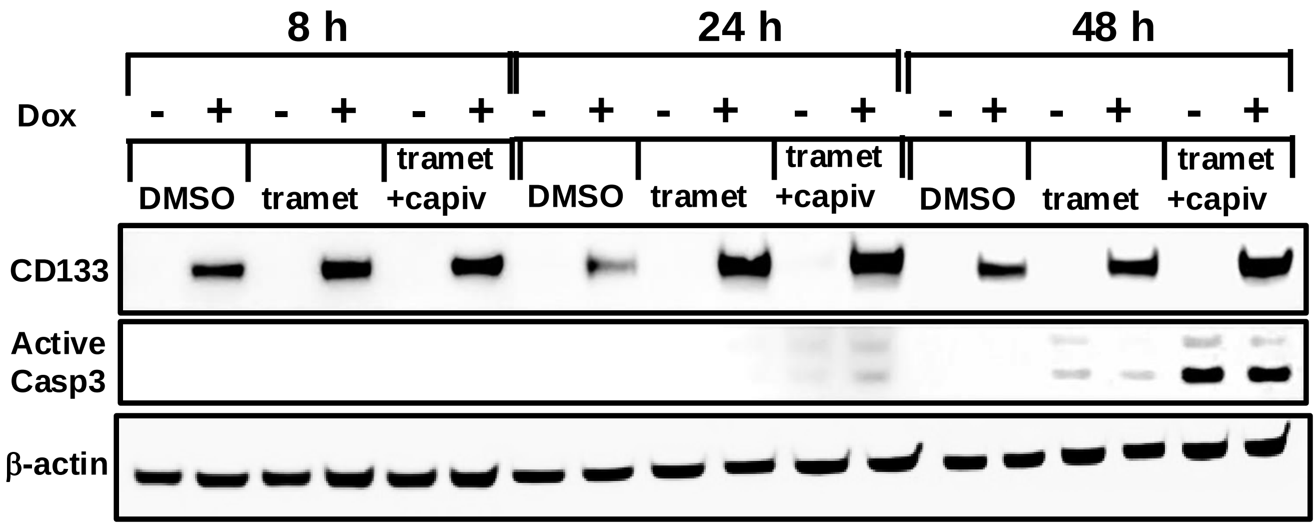

Supplementary Figure S2. Time Course Experiment (of CD133 expression and caspase 3 activation by cleavage in Dox-inducible BAKP cells treated for 8 h, 24 h, or 48 h with vehicle (DMSO), capivasertib, trametinib, or the combination of capivasertib + trametinib. The 48-hour treatment with the combination of capivasertib + trametinib induced the highest activation of caspase 3, an apoptotic marker.

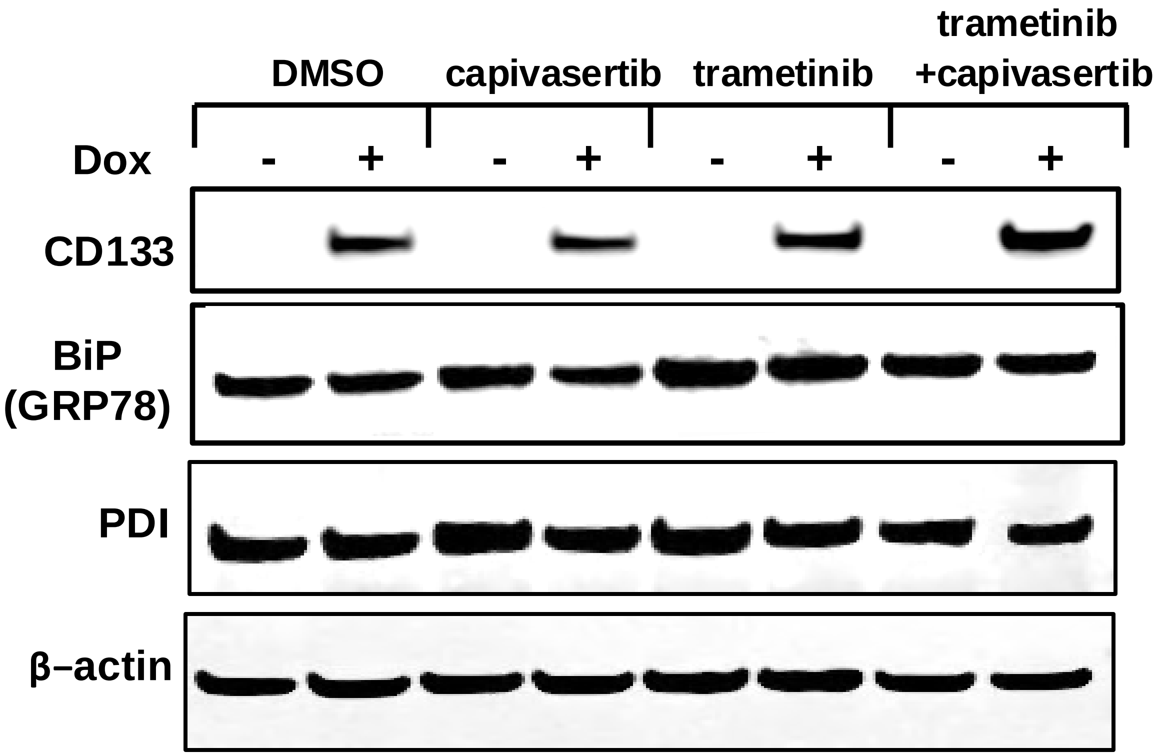

Supplementary Figure S3. Apoptosis induced in BAKP melanoma cells after treatment with trametinib, alone or in combination with capivasertib, is not associated with increased ER stress. Cells were treated with Dox for 24 h to induce CD133 expression, then incubated for 48 h with capivasertib, trametinib, or the combination of the two. Cells were then collected and total cell lysates were subjected to immunoblot analysis with antibodies BiP (GRP78) and PDI, markers of ER stress signaling. Membranes were reprobed with antibodies to β-actin for confirmation of equal protein loading.

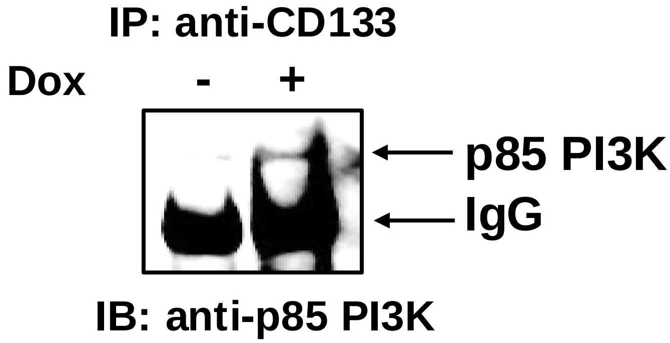

Supplementary Figure S4. Immunoprecipitation experiment verifying the binding of the p85 subunit of PI3K to CD133. BAKP cells were induced to express CD133 by incubation for 24 h with Dox, and cell lysates were extracted and subjected to immunoprecipitation with anti-CD133 bound to beads. Beads were washed, centrifuged down, and then subjected to SDS-PAGE and immunoblot analysis with anti-p85 subunit of PI3K.

Table S1. CI values for Figure 6A XTT Cell Viability Assays

Trametinib Dose*	CI values	Relationship
10	0.08	synergism
10^2	0.43	synergism
10^3	0.49	synergism
10^4	0.6	synergism

*+1 μM capivasertib

Table S2. CI values for Figure 6B Annexin Apoptosis Assays

Trametinib Dose	CI values	Relationship
10	0.93	synergism
10^2	0.71	synergism
10^3	0.68	synergism
10^4	0.55	synergism

*+1 μM capivasertib

Table S3. CI values for Figure 9B Tumor Growth +Dox

Days	CI values	Relationship
3	1.19	antagonism
6	1.03	antagonism
9	0.79	synergism
13	0.43	synergism
16	0.75	synergism
20	0.77	synergism

Table S4. CI values for Figure 9B Tumor Growth -Dox

Days	CI values	Relationship
3	0.5	synergism
6	0.01	synergism
9	0.05	synergism
13	0.35	synergism
16	0.43	synergism
20	0.6	synergism

Supplementary Tables S1 to S4. Combination Index (CI) values were computed to determine synergism, additivity, or antagonism between trametinib and capivasertib *in vitro* (Tables 1, 2) and *in vivo* (Tables 3, 4), where CI<1 = synergism; CI = 1 additivity; CI >1 = antagonism.
